# Bonding and Thermal Cycling Performances of Two (Poly)Aryl–Ether–Ketone (PAEKs) Materials to an Acrylic Denture Base Resin

**DOI:** 10.3390/polym13040543

**Published:** 2021-02-12

**Authors:** Tzu-Yu Peng, Saiji Shimoe, Lih-Jyh Fuh, Chung-Kwei Lin, Dan-Jae Lin, Masato Kaku

**Affiliations:** 1 School of Dentistry, College of Dentistry, China Medical University, No.91, Taichung 404, Taiwan; typ@mail.cmu.edu.tw (T.-Y.P.); ljfuh@mail.cmu.edu.tw (L.-J.F.); 2 Research Center of Digital Oral Science and Technology, College of Oral Medicine, Taipei Medical University, No.250, Wu-Hsing Street, Taipei 110, Taiwan; 3 Department of Anatomy and Functional Restorations, Graduate School of Biomedical and Health Sciences, Hiroshima University, 2-3 Kasumi 1-chome, Minami-ku, Hiroshima 734-8553, Japan; shimoe@hiroshima-u.ac.jp (S.S.); mkaku@hiroshima-u.ac.jp (M.K.); 4 School of Dental Technology, College of Oral Medicine, Taipei Medical University, No.250, Wu-Hsing Street, Taipei 110, Taiwan

**Keywords:** dentistry, prosthodontics, polyetheretherketone, polyetherketoneketone, zirconium oxide, acrylic resin, CAD/CAM, bonding strength, thermal cycling fatigue

## Abstract

Poly(aryl–ether–ketone) materials (PAEKs) are gaining interest in everyday dental practices because of their natural properties. This study aims to analyze the bonding performance of PAEKs to a denture acrylic. Testing materials were pretreated by grinding, sandblasting, and priming prior to polymerization with the denture acrylic. The surface morphologies were observed using a scanning electron microscope and the surface roughness was measured using atomic force microscopy. The shear bond strength (SBS) values were determined after 0 and 2500 thermal cycles. The obtained data were analyzed using a paired samples t-test and Tukey’s honestly significant difference (HSD) test (α = 0.05). The surface characteristics of testing materials after different surface pretreatments showed obvious differences. PAEKs showed lower surface roughness values (0.02–0.03 MPa) than Co-Cr (0.16 MPa) and zirconia (0.22 MPa) after priming and sandblasting treatments (*p* < 0.05). The SBS values of PAEKs (7.60–8.38 MPa) met the clinical requirements suggested by ISO 10477 (5 MPa). Moreover, PAEKs showed significantly lower SBS reductions (*p* < 0.05) after thermal cycling fatigue testing compared to Co-Cr and zirconia. Bonding performance is essential for denture materials, and our results demonstrated that PAEKs possess good resistance to thermal cycling fatigue, which is an advantage in clinical applications. The results imply that PAEKs are potential alternative materials for the removable of prosthetic frameworks.

## 1. Introduction

Dental prostheses are of great significance in maintaining the facial contours and aesthetics of geriatric patients. Along with aging and tooth loss, the bones and soft tissues gradually atrophy. If a patient does not have a dental prosthesis, this will further cause the facial contours to collapse and aging characteristics to become apparent [1]. Dental prostheses are extremely important for geriatric patients and can help maintain somatic function or lead to psychological adaptation [2]. With the importance of aesthetics in contemporary society, aesthetic rehabilitation is gradually increasing [3]. Dental implantology is one of the most eye-catching therapeutic approaches in dentistry nowadays, which not only meets aesthetic considerations but expands the applicability of various types of dental prostheses [4]. However, dental implants are invasive treatments that can cause physical and psychological burdens to the elderly. Additionally, considering health, anatomical, and economic aspects, there are restrictions in applying dental implants to geriatric patients [5]. Moreover, as with increasing oral health awareness, the rate of edentulism has rapidly decreased in the past few decades, with tooth loss occurring later in life. Hence, complete denture treatment in geriatric patients has dropped and the need for removable partial denture (RPD) treatments has increased [6]. RPD treatments are non-invasive treatments to reduce the physical and mental burden, to rehabilitate oral function, and to improve psychological and social adaptability for geriatric patients [7].

The main components of RPD include the denture base resin, artificial teeth, and framework. The denture base resin is mainly composed of polymethyl methacrylate (PMMA), while the choice of artificial teeth will depend on the clinical situation (e.g., ceramic, composite resin, etc.) [8]. Additionally, cobalt–chromium (Co-Cr) alloys are currently the standard materials used in RPD frameworks because of their excellent mechanical properties and well-documented scientific results [9]. Aesthetically, however, components such as the clasps in RPD frameworks are easily exposed, resulting in an unpleasant aesthetic appearance [10]. Additionally, metal alloys may potentially cause allergic reactions or adverse galvanic effects when in contact with saliva [11]. With the development of computer-aided design and manufacturing (CAD/CAM) technology, an increasing number of aesthetic dental materials suitable for RPD frameworks are being introduced in dentistry [12].

The most relevant aesthetic dental material is "zirconia", which possesses good mechanical properties and biocompatibility. Thus, zirconia has been widely used in dentistry for the past decade [12,13]. Among dental applications, yttria-stabilized tetragonal zirconia polycrystal (Y-TZP) and ceria-stabilized tetragonal zirconia–alumina nanocomposite (Ce-TZP/A) are the most used materials [14,15]. In addition, a new polymer material called poly(aryl–ether–ketone) (PAEK) has also been successfully introduced into dentistry in the past few years [16,17,18], and includes polymers such as poly(ether–ether–ketone) (PEEK) and poly(ether–ketone–ketone) (PEKK). PAEKs, in addition to having similar merits as zirconia, also have low density and allow diverse color choices [16,18,19]. Additionally, PAEKs have similar mechanical properties to human bone, with good shock-absorbing properties, and their surfaces are not prone to biofilm formation [18,20,21]. As such, PAEKs have attracted much attention in prosthetics dentistry and epidemiology and are further expected to become the new aesthetic dental material of choice in next-generation treatments.

In previous studies, scholars have tried to apply Y-TZP [22], Ce-TZP/A [23], and PEEK [24] to RPD clasps. The mechanical properties (i.e., fatigue, bending properties) of these materials were tested and the feasibility of applying them to a RPD framework was confirmed. Nevertheless, for the application of aesthetic dental materials to RPD frameworks, critical factors include sufficient mechanical properties and the bonding properties between the materials and the denture acrylic. Studies on zirconia-based materials (Y-TZP and Ce-TZP/A) bonded to denture acrylic have shown that zirconia treated with proper pretreatment methods will provide sufficient bonding strength [25,26]. However, to our knowledge, there are few studies discussing the bonding properties of denture acrylic and PAEKs. 

The aim of this paper is to analyze the bonding strength and durability of PAEKs (PEEK and PEKK) to denture acrylic after proper pretreatment and to further evaluate their clinical applicability. The null hypothesis is that PAEKs have bonding properties with denture acrylic equivalent to those of the Co-Cr alloy or zirconia-based materials (Y-TZP and Ce-TZP/A).

## 2. Materials and Methods

### 2.1. Specimen Preparation and Surface Pretreatment

Five different kinds of dental materials were used in this study. The details of the materials used in this study are provided in Table 1. Disk-shaped specimens (diameter of 10 mm and thickness of 2.5 mm) were prepared for each material using CAD/CAM systems. In total, 300 samples were prepared (*n* = 60 per dental material). All specimens were ground flat with 600-grit silicon carbide abrasive paper (Waterproof Silicon Carbide Abrasive Paper; Kingdom Abrasive Co., Ltd., Taichung, Taiwan), followed by ultrasonic cleaning (RUC-101, REXMED Industries Co., Ltd., Kaohsiung, Taiwan) and air-drying [27]. Subsequently, specimens were randomly distributed into four surface pretreatment conditions: NP group, no pretreatment; SB group, sandblasting pretreatment using 50 μm mean particle size alumina (Al_2_O_3_) particles (Cobra, Renfert GmbH, Hilzingen, Germany); PR group, primer pretreatment by priming with corresponding primer for each material; AP group, pretreatment with both sandblasting and primer.

### 2.2. Surface Roughness and Morphologies

The atomic force microscopy (AFM) was carried out using a scanning probe microscope system (Bruker Dimension Icon VT-1000, Santa Barbara, CA, USA). A silicon probe was selected as the tapping mode to obtain the surface morphologies and the sub-micro scale roughness (Ra) of the surfaces taken from the flattened surfaces under a 50 × 50 μm^2^ area [28]. The representative surfaces were observed using a thermal field emission scanning electron microscope (Thermal FE-SEM) (JEOL JSM-7800F Prime, JEOL Ltd., Tokyo, Japan). The specimens were primarily gilded with platinum under 10 mA/ 25 s (JEOL JEC-3000FC Auto Fine Coater JEOL Ltd., Tokyo, Japan). The specimen chamber was set at a vacuum level of 9.6 × 10^−5^ Pa and the specimens were observed under an accelerating voltage of 3.0 kV using secondary electron mode. Images were captured using the lower electron detector of the FE-SEM system, while magnification strengths of 500× and 2000× were used.

### 2.3. Hydrophilicity (Contact Angle)

The hydrophilicity of the testing specimens (NP group, SB group, PR group, and AP group) was determined using a contact angle analyzer (FTA-125, First Ten Angstroms, Portsmouth, VA, USA). An approximately 10 μL droplet of distilled water was vertically extruded from a 31G needle onto the testing specimens at room temperature and a continuous record charge-coupled device (CCD) was triggered and pictured. A non-spherical fitting approach measured the contact angle of each water drop on the picture. Each reported contact angle was the mean of 10 independent measurements [28].

### 2.4. Bonding Procedure and Thermal Cycling Fatigue Test

For the denture acrylic (SR Triplex Cold, Ivoclar Vivadent AG, Schaan, Liechtenstein) the polymer (polymethyl methacrylate, PMMA) and monomer (methyl methacrylate, MMA) were thoroughly mixed according to manufacturer-recommended application steps and bonded to testing specimens by pouring the resin into a brass mold (diameter, 6.0 mm; height, 2.0 mm). The sample size for the three testing groups (i.e., SB, PR, and AP) of specific dental materials was 20 specimens (*n* = 10 underwent and *n* = 10 did not undergo thermal cycling). After the bonding procedure, all the specimens were dried at 23 °C for 24 h. One-half of the specimens were further exposed to thermal cycling via a hot–cool condition cycle motion system (Chung Chiao Technology Co., Taichung, Taiwan) in de-ionized water for 2500 cycles, at temperatures between 5 °C and 55 °C, with a dwell time of 30 s [27].

### 2.5. Shear Bonding Strength (SBS) Testing

A desktop testing machine (JSV-H1000, Japan Instrumentation System Co., Ltd, Nara, Japan) was used to perform SBS testing on specimens that underwent and did not undergo the thermal cycling fatigue test (*n* = 10). The SBS testing conditions were set at the constant crosshead speed of 1 mm/min, in accordance with the standard ISO 10477: 2018 [29]. Shear force was applied to the adhesive interface until fracturing occurred. Load values of the debonded dental materials and denture acrylic were measured, and SBS values were calculated using the following formula:

*SBS (*MPa*) = The force at fracture (*N*)/bonding area of the specimen* (mm^2^) (1)

### 2.6. Analysis of Residual Adhesives

After SBS testing, the debonded surface was observed with an optical microscope under 20× (Olympus BX40, Olympus Optical Co. Ltd., Tokyo, Japan) to define the failure mode. The failure modes were classified into adhesive failure, cohesive failure, and a mixture of adhesive and cohesive failures (mixed failure). The structures of the residual adhesives on the surface were evaluated using a Thermal FE-SEM instrument (JEOL JSM-7800F, JEOL Ltd., Tokyo, Japan). Magnification strengths of 500× and 2000× were used for comparison with observations obtained using optical microscopy [27].

### 2.7. Statistic Analysis

The normality of the distribution was primarily analyzed using the Shapiro–Wilk test, while the homogeneity of variance was confirmed via Levene’s test. Multiple comparisons of different material groups (Co-Cr alloy, Y-TZP, Ce-TZP/A, PEEK, PEKK) were analyzed using one-way analysis of variance (ANOVA) with Tukey’s honestly significant difference (HSD) tests. Statistical comparison of differences between pretreatment conditions for each group was carried out using the paired samples *t*-test. All statistical analyses were performed using IBM SPSS statistical software (SPSS version 19; IBM, Armonk, NY, USA). The significant differences were set to *p* < 0.05 if not specified.

## 3. Results

### 3.1. Characterizations of the Material Surfaces

#### 3.1.1. Surface Roughness and Topography

**Figure 1:**
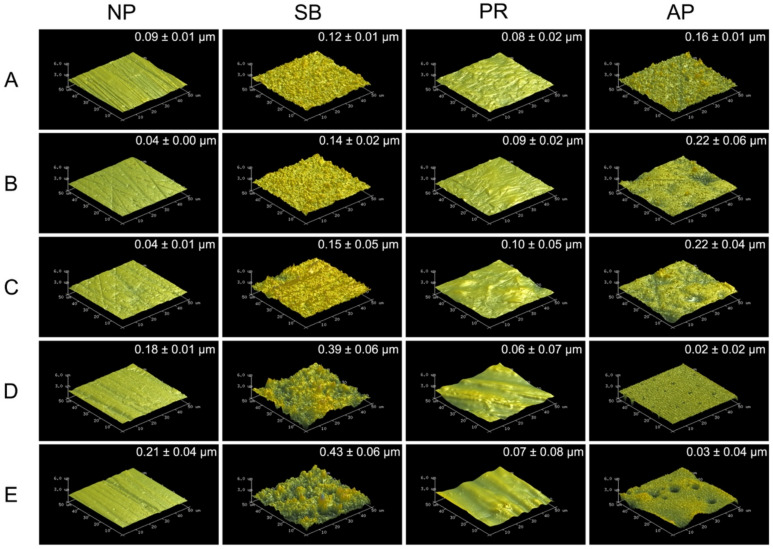
The atomic force microscopy (AFM) 50 × 50 μm^2^ images for each material and surface pretreatment condition: (**A**) Co-Cr; (**B**) Y-TZP; (**C**) Ce-TZP/A; (**D**) PEEK; (**E**) PEKK. The column was the surface pretreatment condition (NP, non-treatment; SB, sandblasting pretreatment using 50 μm mean particle size alumina (Al_2_O_3_) particles; PR, primer; AP, both sandblasting and primer). The sub-micro scale roughness (*Ra*) values of the flattened surfaces are shown in each image (means ± SD, *n* = 5).

Figure 1 shows 50 × 50 μm^2^ AFM images of various types of testing materials after different surface pretreatment conditions. The sub-micro scale roughness (Ra) is shown in the upper right of each AFM image. Zirconia-based materials (Y-TZP and Ce-TZP/A) had similar Ra and surface characterizations as Co-Cr alloy, while PEEK and PEKK also had comparable Ra and surface characterizations. As expected, the surface roughness values of each specimen were all significantly increased after sandblasting. Within the NP and SB groups, PAEKs had significantly higher Ra values (0.18~0.21 μm) than other materials (*p* < 0.05). However, the Ra of PAEKs decreased after undergoing priming (PR or AP group), which was opposite to what was observed in Co-Cr alloy and zirconia-based materials, for which the Ra of PR and AP groups were slightly higher than that of NP and SB groups, respectively. Regarding the AP group, Co-Cr alloy and zirconia-based materials showed the highest Ra among all test conditions. Nevertheless, in PAEK, Ra ranged from the micrometer scale down to the nanometer scale (PEEK: 0.02 ± 0.02 μm and PEKK: 0.03 ± 0.04 μm).

#### 3.1.2. Surface Morphology

**Figure 2:**
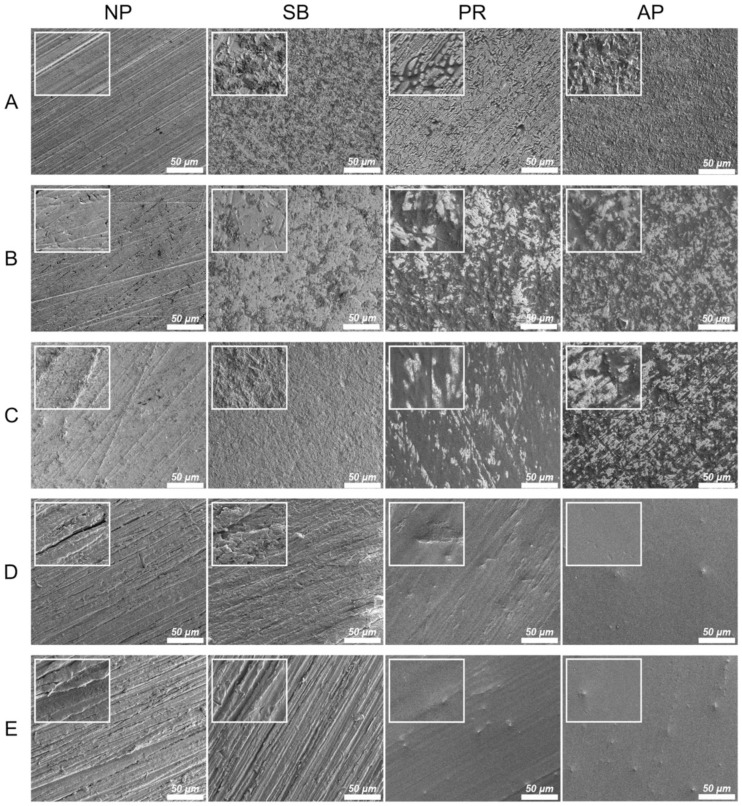
The FE-SEM surface morphologies for each material and surface pretreatment condition: (**A**) Co-Cr; (**B**) Y-TZP; (**C**) Ce-TZP/A; (**D**) PEEK; (**E**) PEKK. The column was the surface pretreatment condition (NP, non-treatment; SB, sandblasting pretreatment using 50 μm mean particle size alumina (Al_2_O_3_) particles; PR, primer; AP, both sandblasting and primer). The scale bar is 50 μm (magnifications of 500), and the width of the small, enlarged picture is equal to 2.5 μm (magnifications of 2000).

Figure 2 shows the thermal SEM (FE-SEM) surface morphology images for all testing conditions. In the Co-Cr alloy and zirconia-based materials, the NP group exhibited scratches characteristic of those caused by silicon carbide abrasive paper, while the surface of the SB group was uneven and rough. Chemical erosion characteristics were observed on the material surfaces of the PR group, especially in areas with scratches characteristic of the NP group. The AP group simultaneously showed unevenness caused by sandblasting and chemical erosion characteristics caused by priming. In PAEKs, the NP group showed scratches similar to other materials. However, the SB group had surface morphologies analogous to those of the NP group. However, after being primed (PR group), the surface became smoother and did not have obvious unevenness or roughness. Note that in the AP group, small-swelling surface morphologies were found, and a similar aperture-shaped construction can also be found in the AFM images (Figure 1).

#### 3.1.3. Hydrophilicity

**Figure 3:**
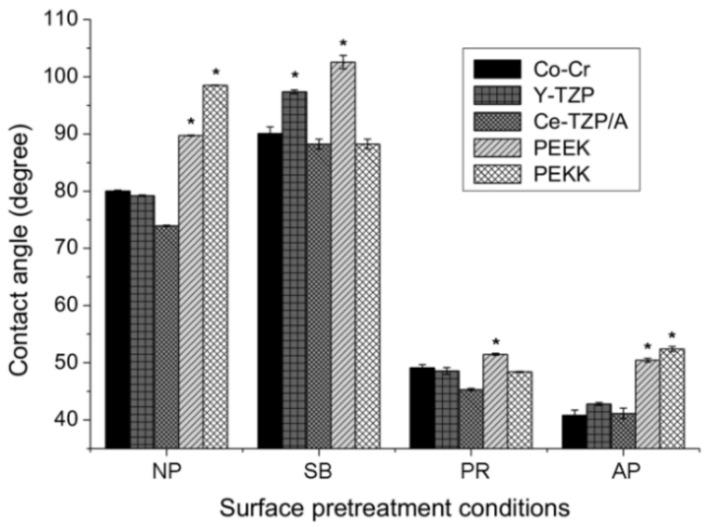
The contact angles of each material (Co-Cr, Y-TZP, Ce-TZP/A, PEEK, and PEKK) and surface pretreatment condition (NP, non-pretreatment; SB, sandblasting pretreatment using 50 μm mean particle size alumina (Al_2_O_3_) particles; PR, primer; AP, both sandblasting and primer). Note: * significant different to other materials (*p* < 0.05).

The levels of hydrophilicity of each material and each different surface pretreatment condition were compared in Figure 3. Without surface pretreatment (NP group), the PAEKs showed a contact angle that exceeded 90°, which indicates super-hydrophobicity when compared to Co-Cr alloy and zirconia-based materials (*p* < 0.05). After surface pretreatment, in the PR group, the contact angles for all materials significantly decreased (*p* < 0.05) compared to the NP group. After sandblasting (SB group), the contact angles significantly increased in most materials, except PEKK (88.2°). PEEK had the highest contact angle of 102.5° among all test conditions. In the AP group, Co-Cr alloy and zirconia-based materials showed the lowest contact angles among all test conditions. However, the contact angle of PAEKs in the AP group was comparable to that of the PR group and exhibited significantly less hydrophilicity, with a value of approximately 50° compared to Co-Cr alloy and zirconia-based materials (*p* < 0.05).

### 3.2. Bonding Properties

#### 3.2.1. Shear Bond Strength (SBS)

Table 2 summarizes the results of the SBS tests. Regardless of the material, the AP group showed the highest SBS. In Co-Cr alloy and zirconia-based materials, the SBS result for the PR group was significantly higher than that of the SB group (*p* < 0.05), while PAEKs had similar SBS results for both PR and SB groups. When considering the various surface pretreatment conditions, there were no significant differences between various materials for the SB group. However, PAEKs and the Co-Cr alloy had higher SBS values than zirconia-based materials. Within the PR group, Co-Cr alloy and zirconia-based materials had comparable SBS values and were higher than PAEKs. The result for the AP group showed that the SBS values of PAEKs were lower than those of the Co-Cr alloy and zirconia-based materials, but nevertheless were consistent with the clinical guidelines of ISO 10477 (>5.0 MPa).

#### 3.2.2. SBS after Thermal Cycling Fatigue Test

Regarding the SBS results (Table 2) for the AP group, the SBS values for zirconia-based materials were reduced significantly and were lower than the ISO 10477 standard of 5 MPa. The Co-Cr alloy had the highest SBS value (7.14 ± 2.05 MPa), PAEKs had the lowest SBS reduction, while Co-Cr alloy and PAEKs (PEEK: 5.64 ± 0.64 MPa; PEKK: 5.77 ± 0.61 MPa) all exceeded 5 MPa. Outside of the AP group, only the Co-Cr alloy in the PR group (7.01 ± 0.84 MPa) had a larger SBS than 5 MPa. Table 2 lists the reductions of SBS after the thermal cycling fatigue test. The SBS values of all materials were reduced after the thermal cycling fatigue test. Irrespective of the material, the reductions in the PR group were relatively small (zirconia-based materials: SB > AP ≈ PR; Co-Cr alloy: SB > AP > PR; PAEKs: AP > SB > PR). The reductions in zirconia-based materials were the highest (> 60%), while the SBS under the SB group declined to 0 MPa. The remaining reductions in descending order were Co-Cr alloy > PEKK > PEEK. For PEEK materials, regardless of the surface pretreatment conditions or whether the samples underwent the thermal cycling fatigue test or not, the SBS results showed no significant differences.

#### 3.2.3. Mode of Failure

**Figure 4:**
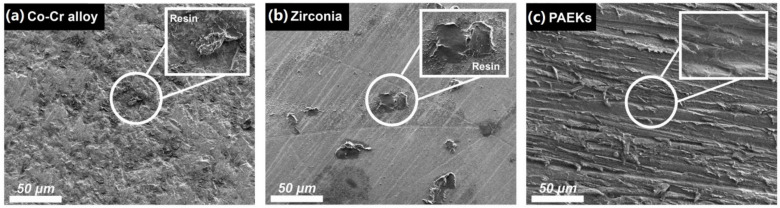
The representative debonded surface morphologies of each group of materials. The FE-SEM image (the magnification was 500× and the scale bar was 50 μm) of (**a**) the Co-Cr alloy (AP group, pretreatment with both sandblast and primer), (**b**) zirconia-based ceramic (PR group, primer pretreatment by priming with primer), and (**c**) (Poly)aryl–ether–ketone materials (PAEKs) (AP group). Residual denture acrylic debris was found in the Co-Cr alloy and zirconia-based ceramic, as shown in the magnified pictures, while the debonded surfaces of the PAEKs shown no residuals.

Table 2 illustrates the classification of failure modes after SBS testing. Adhesive failure modes were predominant in all materials irrespective of whether the thermal cycling fatigue test was performed and of the surface pretreatment conditions. Residual denture acrylic can be observed in the representative FE-SEM images (Figure 4) of the debonding interfaces of the Co-Cr alloy and zirconia-based materials (the combination of cohesion and adhesion failure), while the debonding interfaces of PAEKs showed no residual denture acrylic (adhesion failure).

**Figure 5:**
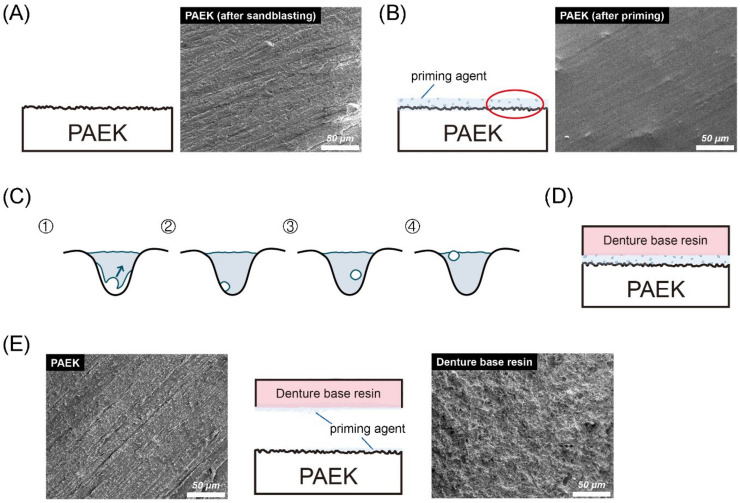
Bonding and debonding schematic diagram and FE-SEM images (the magnification was 500× and the scale bar was 50 μm) of (poly)aryl–ether–ketone (PAEK) materials. Firstly, the sandblasted surfaces of PAEKs retained their scratch characteristics (**A**). After the priming process, swelling bubbles were found on the surfaces of PAEKs (**B**). (**C**) The cavities shown in the figure are an enlarged schematic diagram of the irregular holes created by sandblasting. The volume shrinkage that occurred during the photopolymerization process was accompanied by the generation of bubbles (①). During the photopolymerization process, the bubbles would gradually move to the primer surface (②→③). After the photopolymerization was completed, if bubbles moved onto the primer surface (④), these would become swelling bubbles; if the bubbles adhered to the substrate wall (③), the contact area between the priming agent and PAEK would reduce. Schematic diagram of denture acrylic bonded to PAEKs is shown in (**D**). After the SBS test, the denture acrylic was debonded from the PAEK surface. According to the FE-SEM image (**E**), the appearance of the PAEK side (base material; left FE-SEM images) was the same as the sandblasting surface (**A**), while the denture’s acrylic side (adhesion; right FE-SEM images) was rough and uneven.

Figure 5 shows the bonding and debonding schematic diagram of PAEKs (AP group). As mentioned before (Figure 1 and Figure 2), PAEKs still showed characteristic scratches after sandblasting (Figure 5A), while the surfaces became smoother with a few bubbles after priming (Figure 5B,C). The schematic diagram of denture acrylic bonded to PAEKs is shown in Figure 5D. After debonding (Figure 5E), it could be observed that PAEKs (substrate) showed scratches characteristic of the SB group, while denture acrylic (adherent) showed an irregular rough surface.

## 4. Discussion

The present study evaluated the shear bonding strength (SBS) and bonding durability of poly(aryl–ether–ketone) materials (PAEKs) to a denture acrylic using a Co-Cr alloy and two zirconia-based materials for comparison. The effects of surface pretreatments on the bonding characteristics were also investigated. Our results indicated that when following recommended surface pretreatment (AP group) and under static SBS test conditions, the bonding strengths between denture acrylic and PAEKs were relatively lower than those of Co-Cr alloy or zirconia-based materials (Table 2). However, after thermal cycling fatigue, the reductions in bonding strength of PAEKs were remarkably lower than the other two groups, although the SBS was still lower than that of the Co-Cr alloy. However, the SBS values between denture resin and PAEKs were apparently higher than those of zirconia-based materials (Table 2).

It is known that the bonding mechanism between adherent and substrate materials includes chemical preprocessing (i.e., priming, acid etching, etc.) [30,31], mechanical preprocessing (i.e., sandblasting, lasering) [32,33], or a combination of both methods. Within the present study, all testing materials were treated with sandblasting pretreatment (SB group), priming pretreatment (PR group), or a combination pretreatment of sandblasting and priming (AP group). From the AFM (Figure 1) and FE-SEM (Figure 2) images, it can be observed that the surface topographies of Co-Cr alloy and zirconia-based materials in SB group were similar, but PAEKs were apparently different from other materials. The higher roughness of PAEKs after erosion from sandblasting was considered to indicate that PAEKs are more flexible and have a lower elastic modulus and hardness [16,17]. The PR and AP groups, which underwent priming pretreatment, had their surface roughness compared before and after priming (NP→PR group; SB group→AP group). It was observed that the surface roughness of Co-Cr alloy and zirconia-based materials had slightly increased after priming pretreatment, however the difference was not significant. Conversely, the surface roughness of PAEKs decreased after priming pretreatment (Figure 1). Additionally, it was important to note that in the AP group of PAEKs, pinhole-like surface cavities were observed in the AFM images (Figure 1). This structure (small-swelling surface morphologies) was also found in FE-SEM images (Figure 2).

The author speculated that the difference in structure was due to the priming agent used in PAEKs being composed of MMA [34]. Due to the high viscosity of the PAEKs’ priming agent, a thick primer layer was formed after photopolymerization. Thus, the concave–convex surface generated by sandblasting was filled and reduced the surface roughness. Conversely, the priming agent used for the Co-Cr alloy and zirconia-based materials had high flowability and good wettability after drying, so that a thin film formed without reducing the surface roughness [25,30]. Additionally, the polymerization of MMA to form PMMA results in significant polymerization shrinkage (21 vol%) [35]. Even when the polymer-to-monomer ratio was adjusted to approximately 3:1, the volume shrinkage rate was still about 7% [36]. During photopolymerization, the PAEKs’ priming agent largely shrunk and generated bubbles. When the polymerization was completed, the bubbles that did not float to the surface of the primer layer became stuck in the primer layer, and many small swelling bubbles were generated on the surface. (Figure 5B,C). However, the primer layer was thin, so the cavities could be observed when using AFM to analyze the surface sub-nanometer structure. Nonetheless, the mechanism still needs to be analyzed in future experiments.

Different surface pretreatment methods led to inverse SBS results (Table 2). The aim of sandblasting (SB group) was to increase the surface roughness of substrate materials and increase the mechanical interlocking of denture acrylic and substrate materials, further increasing the bonding durability [32,33]. When compared to the PR group (8.36 ± 2.61–9.76 ± 1.17 MPa) and the AP group (11.80 ± 4.63–12.47 ± 4.84 MPa), the Co-Cr alloy and zirconia-based materials showed the lowest SBS values in the SB group (1.83 ± 0.48–4.62 ± 1.24 MPa). This is due to the Co-Cr alloy and zirconia having higher hardness values, causing the irregularities formed from sandblasting to be superficial [27,37]. PAEKs have lower hardness values, so they exhibit relatively higher SBS values. Shimoe et al. analyzed the sample after sandblasting with alumina (Al_2_O_3_) particles through X-ray photoelectron spectroscopy, and the experimental results confirmed that Al_2_O_3_ particles were mechanically attached to the surface of the material after sandblasting. The present experimental results also showed that sandblasting increased the surface hydrophobicity of Co-Cr alloy and zirconia-based materials (Figure 3), presumably because of the contamination from the sandblasting process. 

The priming agent containing 10-methacryloyloxydecyl dihydrogen phosphate (MDP) was most recommended for Co-Cr alloy and zirconia-based materials due to its bifunctional adhesive monomers (hydrophobic methacrylate and hydrophilic phosphate terminal end), which can copolymerize resin monomers and chemically bind to oxides [30,38]. The results of this study confirmed the positive effects of priming agents on Co-Cr alloy and zirconia-based materials. Additionally, after priming pretreatment (both PR group and AP group), the surface hydrophilicity of the Co-Cr alloy and zirconia-based materials increased (Figure 3). This result was expected due to the molecular structure of the MDP monomer being hydrophilic [39,40]. The literature suggests applying priming agents containing MMA to PAEKs due to the dimethacrylate monomers providing a connection to the resins, with two carboxyl groups acting as binding sites [34,38]. Nevertheless, compared to other testing materials and the SB group, the SBS values of PAEKs did not improve, even after priming pretreatment. According to the requirements of ISO 10477, the minimum shear bond strength value at the interface of resin-based materials and substrates that is considered acceptable for clinical use is 5 MPa [29]. However, when the PAEKs only underwent chemical bonding, the bonding strength did not achieve the clinical criteria (3.82 ± 0.32–3.78 ± 0.63 MPa). Therefore, mechanical bonding (AP group) needs to be added to improve the bonding properties (7.60 ± 1.99–8.38 ± 1.92 MPa). In order to increase the adhesive performance (i.e., bonding strength) to achieve the clinical criteria, the priming agent for PAEKs will need to be improved in the future.

When undergoing the thermal cycling fatigue test, Co-Cr alloy and zirconia-based materials exhibited significant SBS reduction (Table 2). Some scholars have pointed out similar results and speculated that this is caused by the differences in the thermal expansion coefficients of the materials [25,26,33]. From the debonding results, it was found that adhesive failure was predominant (Table 2) and some specimens left behind residual resins on the substrate surfaces (Figure 4). However, regardless of the surface pretreatment conditions used for PEEK, the SBS was not significantly decreased (reduction<26%). The reason is that the thermal expansion coefficient of PEEK was similar to that of denture acrylic, so the temperature effect was not reflected in the SBS values. These results could further indicate that PEEK has the best bonding durability among the five testing materials, especially after long-term application. From the debonding results, it was found that PAEKs all experienced adhesive failure (Table 2). The interface of the debonded materials was observed using FE-SEM (Figure 5E). Because the priming agent was applied on the rough surface (after sandblasting) of the PAEK (Figure 5A), the surface of the debonded denture resin was also uneven. Many plastic deformation dimples were found on the debonded denture acrylic’s surface (Figure 5E, right FE-SEM images), which were considered the appearance of the remaining deformed priming agent. The debonded surfaces of PAEKs (substrate; Figure 5E left FE-SEM images) were the same as the sandblasted surfaces (Figure 5A).

As described above, the priming agent for PAEKs, which contained MMA, shrunk obviously during the photopolymerization and further generated bubbles. When bubbles adhering to scratches or textures produced via silicon carbide abrasive paper or sandblasting formed, the contact area between the priming agent and the substrate material decreased, thereby reducing the bonding strength (Figure 5C). In addition, the small bubbles became stress concentration sites and weakened the bonding interface (Figure 5C). Based on these testing results, it is apparent that the adherent properties between the priming agent and denture acrylic were better than those between the priming agent and PAEKs. Thus, the priming agent tightly bonded to denture acrylic after shearing force was applied and the priming agent adhered to the adherent rather than to the substrate (adhesion failure). Therefore, there is still a need to improve the priming agent applied to PAEKs in the future to improve the bonding strength between the primer and the PAEK substrate.

Regardless of the material, the combination of sandblasting and priming pretreatment (AP group) resulted in the best SBS, which inferred that the use of sandblasting and priming at the same time would be the optimal pretreatment method for denture acrylic used for PAEKs. In particular, the author observed that the SBS results of sandblasting and priming pretreatment methods were subject to additivity. Thus, multiple surface pretreatments on the material might improve the bonding properties. This point should be evaluated in clinical operation. Before the thermal cycling fatigue test, the SBS values for Co-Cr alloy and zirconia-based materials exceeded 5 MPa (in line with ISO10477), except for in the SB group. After the thermal cycling fatigue test, the SBS results for Co-Cr alloy were higher than 5 MPa, except in the SB group (2.49 ± 0.59 MPa). However, zirconia-based materials all dropped to 5 MPa. When considering the bonding durability, zirconia-based materials were not suitable for use in the removable partial denture (RPD) framework. Regardless of whether the thermal cycling fatigue test was performed, SBS values of PAEKs exceeded 5 MPa in the AP group (Table 2), and the reduction of SBS was also significantly lower than that of other testing materials (Table 2). 

The literature demonstrates the application of PAEK with good anti-adhesive properties to oral bacteria. However, in PAEK frameworks with oral bacterial colonies, whether biofilms will form and whether inflammatory reactions will occur still remain unclear. This issue should be confirmed through further research to clarify the relevant effects between removable prosthetic frameworks and oral microbiology, so that the long-term usage feasibility of application on removable prosthetic frameworks can be determined. Note that the superior flexible properties of PAEK materials would relate to the life span of the PAEK prosthesis (i.e., relative lower resistance to force). Peng et al. [24] reported that the deformation was not significantly different to Co-Cr alloy after 15,000 cycles (equal to 5 years clinical usage) when adjusting the thickness or width of the framework. Hence, the PAEK framework successfully enhanced the bite force and fulfilled the long-term requirements. Additionally, it is known that PAEK, with its excellent fatigue resistance, abrasion resistance, and hydrolysis resistance, is highly stable in the oral cavity and does not experience qualitative changes [21].

A comprehensive consideration of the present investigation shows that PAEKs can be used as potential alternative aesthetic materials for RPD frameworks. However, some additional variables could alter the results of the present report. For instance, mechanical agents such as occlusal wear and toothbrushing could have significant effects on materials surfaces, thus influencing the results of the present study in terms of roughness, morphology, and bond strength results. Further studies are needed in order to encompass these important factors.

## 5. Conclusions

Priming combined with sandblasting (AP group) was the optimal surface pretreatment method for poly(aryl–ether–ketone) materials (PAEKs). After this pretreatment, the PAEKs had sufficient shear bond strength (SBS) with denture acrylic and fulfilled the clinical guidelines of ISO 10477 (>5.0 MPa). Compared with Co-Cr alloy and zirconia-based materials, the PAEKs showed significantly lower SBS reduction levels after thermal cycling fatigue testing, indicating that the PAEKs possessed adequate bonding durability. However, the bonding performance of the PAEKs still has room for improvement, and therefore the priming agent for PAEKs will need to be improved in the future to enhance the bonding strength and durability between priming agents and PAEK substrates. The PAEKs are natural materials with good biocompatibility and shock absorption and are aesthetically pleasing. In combination with the present feasibility study, the PAEKs are evaluated as potential alternative materials for removable prosthetic frameworks.

## Figures and Tables

**Table 1:** Material assessed in the present study.

Groups	Materials (Dentification)	Main Composition *	Manufactures (Batch No.)
Co-Cr	Framework: Wironit Extra-hard	Co, Cr, Mo	BEGO, Bremen, Germany (132300912)
	Primer: Clearfil Ceramic Primer Plus	MDP, silane, ethanol	Kuraray Medical Inc., Tokyo, Japan (AV0049)
Y-TZP	Framework: TZ-3YB-E	ZrO_2_, Y_2_O_3_	Tosoh Corporation, Tokyo, Japan (S305374B)
	Primer: Clearfil Ceramic Primer Plus	MDP, silane, ethanol	Kuraray Medical Inc., Tokyo, Japan (AV0049)
Ce-TZP/A	Framework: Cpro NANO Zirconia	ZrO_2_, Al_2_O_3_, CeO_2_	YAMAKIN Co., Ltd., Osaka, Japan (CD90011076J)
	Primer: Clearfil Ceramic Primer Plus	MDP, silane, ethanol	Kuraray Medical Inc., Tokyo, Japan (AV0049)
PEEK	Framework: VESTAKEEP	poly(ether–ether–ketone)	Evonik Japan Co., Tokyo, Japan (57781699)
	Primer: visio. link	PETIA, MMA, photo initiators	Bredent, Senden, Germany (700388)
PEKK	Framework: Pekkton ivory	poly(ether–ketone–ketone)	Cendres+Métaux SA, Biel/Bienne, Switzerland (335773)
	Primer: visio. link	PETIA, MMA, photo initiators	Bredent, Senden, Germany (700388)

* According to the information provided by the manufacturer. Groups: Co-Cr, cobalt-chromium alloy; Y-TZP, yttria-stabilized tetragonal zirconia polycrystal; Ce-TZP/A, ceria-stabilized tetragonal zirconia–alumina nanocomposite; PAEKs, poly(aryl–ether–ketone); PEEK, poly(ether–ether–ketone); PEKK, poly(ether–ketone–ketone). Framework, product name of the substrate materials, Primer, product name of the priming agent used in each group. MMA, methyl methacrylate; MDP, 10-methacryloyloxydecyl dihydrogen phosphate; PETIA, pentaerythritol triacrylate.

**Table 2:** Mean shear bond strength (MPa), failure mode, and reduction values for each testing group after different surface pretreatment conditions.

Condition	Group	0 Thermal Cycle		2500 Thermal Cycles		Reduction
		Mean ± SD	Failure Mode A/AC/C	Mean ± SD	Failure Mode A/AC/C	
SB	Co-Cr	4.62 ± 1.24 *^a, b^*	10/0/0	2.49 ± 0.59 ^*α*^	10/0/0	46.08% *
	Y-TZP	2.00 ± 0.44 *^a^*	10/0/0	0.00 ± 0.00 ^*β*^	10/0/0	100.00% *
	Ce-TZP/A	1.83 ± 0.48 *^a^*	10/0/0	0.00 ± 0.00 ^*β*^	10/0/0	100.00% *
	PEEK	3.07 ± 0.45 *^a^*	10/0/0	2.76 ± 0.45 ^*α*^	10/0/0	10.09%
	PEKK	4.75 ± 1.15 ^*a, b*^	10/0/0	3.35 ± 0.72 ^*α, γ*^	10/0/0	29.43% *
PR	Co-Cr	9.76 ± 1.17 ^*c, d, e, h*^	8/2/0	7.01 ± 0.84 ^*δ*^	10/0/0	28.24% *
	Y-TZP	9.50 ± 3.52 ^*c, d, e, h*^	9/1/0	3.78 ± 1.77 ^*α, ε,*^ *^ϝ^*	10/0/0	60.27% *
	Ce-TZP/A	8.36 ± 2.61 ^*f, g, h*^	9/1/0	3.02 ± 1.58 ^*α,*^ *^ϝ^*	9/1/0	63.92% *
	PEEK	3.82 ± 0.32 ^*a*^	10/0/0	3.50 ± 1.19 ^*α,*^ *^ϝ, η^*	10/0/0	8.39%
	PEKK	3.78 ± 0.63 ^*a*^	10/0/0	2.96 ± 1.20 *^α,^^ϝ^*	10/0/0	12.60%
AP	Co-Cr	11.73 ± 1.82 ^*c, d, f*^	8/2/0	7.14 ± 2.05 ^*δ*^	10/0/0	39.14% *
	Y-TZP	12.47 ± 4.84 ^*c*^	8/2/0	4.93 ± 1.28 ^*γ, ε, η, ω*^	10/0/0	60.43% *
	Ce-TZP/A	11.80 ± 4.63 ^*c, d, f*^	8/2/0	4.54 ± 1.47 ^*γ,*^ *^ϝ, ω^*	10/0/0	61.49% *
	PEEK	7.60 ± 1.99 ^*b, e, g, i*^	10/0/0	5.64 ± 0.64 ^*δ, ω*^	10/0/0	25.86%
	PEKK	8.38 ± 1.92 ^*d, f, i*^	10/0/0	5.77 ± 0.61 ^*δ, ω*^	10/0/0	31.11% *

Conditions (surface pretreatment condition): SB, sandblasting pretreatment using 50 μm mean particle size alumina (Al_2_O_3_) particles; PR, primer pretreatment by priming with primer; AP, pretreatment with both sandblast and primer. Group: five kinds of adherent materials. SD, standard deviation. Within the same column, different letters *(a, b, c, d, e, f, g, h, i) (α, β, γ, δ, ε, ϝ, η, ω)* indicate groups that are statistically different (*p* < 0.05). Failure modes: A, adhesive failure; AC, combination of cohesive and adhesive failure; C, cohesive failure. Reduction: comparison of shear bond strength in groups that underwent (2500 thermal cycles) and did not undergo (0 thermal cycle) thermal cycling testing. Note: * Significant difference was based on paired samples *t*-test (*p* < 0.05) and analysis of the reduction between specimens that underwent and did not undergo thermal cycling testing.

## Data Availability

None.
